# Deep Residual Convolutional Neural Networks for Brain–Computer Interface to Visualize Neural Processing of Hand Movements in the Human Brain

**DOI:** 10.3389/fncom.2022.882290

**Published:** 2022-05-20

**Authors:** Yosuke Fujiwara, Junichi Ushiba

**Affiliations:** ^1^Graduate School of Science and Technology, Keio University, Yokohama, Japan; ^2^Information Services International-Dentsu, Ltd., Tokyo, Japan; ^3^Faculty of Science and Technology, Keio University, Yokohama, Japan

**Keywords:** deep learning, brain-computer interface, Grad-CAM, electroencephalography, deep residual convolutional neural networks (CNN)

## Abstract

Concomitant with the development of deep learning, brain–computer interface (BCI) decoding technology has been rapidly evolving. Convolutional neural networks (CNNs), which are generally used as electroencephalography (EEG) classification models, are often deployed in BCI prototypes to improve the estimation accuracy of a participant's brain activity. However, because most BCI models are trained, validated, and tested *via* within-subject cross-validation and there is no corresponding generalization model, their applicability to unknown participants is not guaranteed. In this study, to facilitate the generalization of BCI model performance to unknown participants, we trained a model comprising multiple layers of residual CNNs and visualized the reasons for BCI classification to reveal the location and timing of neural activities that contribute to classification. Specifically, to develop a BCI that can distinguish between rest, left-hand movement, and right-hand movement tasks with high accuracy, we created multilayers of CNNs, inserted residual networks into the multilayers, and used a larger dataset than in previous studies. The constructed model was analyzed with gradient-class activation mapping (Grad-CAM). We evaluated the developed model *via* subject cross-validation and found that it achieved significantly improved accuracy (85.69 ± 1.10%) compared with conventional models or without residual networks. Grad-CAM analysis of the classification of cases in which our model produced correct answers showed localized activity near the premotor cortex. These results confirm the effectiveness of inserting residual networks into CNNs for tuning BCI. Further, they suggest that recording EEG signals over the premotor cortex and some other areas contributes to high classification accuracy.

## Introduction

Brain–computer interface (BCI) decoding techniques have been rapidly evolving in the recent years. Electromyogram (EMG) can be decoded from local field potential (LFP) (Krasoulis et al., 2014), and listened speech can be decoded from electrocorticogram (ECoG) (Pasley et al., 2012; Kubanek et al., 2013; Martin et al., 2018). However, for BCI decoding from electroencephalography (EEG), although the time and spatial resolutions are lower than those of LFP and ECoG, there are ongoing attempts at task classification, such as estimating whether the participant is resting, moving the right or left hand, or moving the foot (Wolpaw et al., 2000; Pfurtscheller et al., 2005; LaFleur et al., 2013; Aghaei et al., 2016). In these BCIs, the methods to convert waveforms, which are divided into time windows or frequency domain features using techniques such as fast Fourier transform (FFT) (Cooley and Tukey, 1965; Welch, 1967), or features using a specific frequency band power, have been used to classify the target task *via* advanced machine learning (Aler et al., 2010a,b; Thang and Temiyasathit, 2014).

A method utilizing common spatial pattern (CSP), which is often used in BCI, has been proposed to improve the accuracy of multiclass classification of motor imagery by utilizing the important clusters in the CSP feature set (Zhang et al., 2021). Further, a method combining CSP and support vector machine (SVM) with regularization has also been shown to improve the classification accuracy of motor imagery through sparse optimization (Jiao et al., 2020). The recent methods with task-related component analysis (TRCA) and canonical correlation patterns (CCP) (Duan et al., 2021) have also been proposed. These methods combine filter banks and SVM to classify between pre-movement and resting states with high accuracy (Jia et al., 2022a). An improved method for multiclass pre-movement classification by optimization with both filter bank and time window selection has also been proposed (Jia et al., 2022b).

Some BCIs have also been applied to rehabilitation using *ad hoc* handcrafted features based on medical or physiological findings instead of advanced machine learning (Shindo et al., 2011; Ramos-Murguialday et al., 2013; Ang et al., 2014; Frolov et al., 2017; Ibáñez et al., 2017; Biasiucci et al., 2018). EEG-based BCI has great potential for medical or everyday device applications owing to its non-invasive property and capability to decode the brain with an inexpensive device compared to functional magnetic resonance imaging (fMRI) (Miyawaki et al., 2008; Naselaris et al., 2011; Nishimoto et al., 2011; Shen et al., 2019a,b). However, because its time and spatial resolutions are lower than those of LFP and ECoG, the decoding accuracy tends to be lower, and complicated decoding is not as effective as in LFP and ECoG.

Recently, with the development of deep learning technologies such as convolutional neural networks (CNNs), including EEGNet, Deep-ConvNet, and Shallow-ConvNet, such issues are gradually being overcome (Sakhavi et al., 2015, 2018; Schirrmeister et al., 2017; Zhang et al., 2017; Lawhern et al., 2018). Yang et al. (2015) reported improved classification accuracy for motor imagery within-subject validation using CNN combined with CSP. Dai et al. (2019) also reported that a method combining CNN and variational autoencoder (VAE) improved the classification accuracy of motor imagery. In addition, there is currently the prospect of classifying even end-to-end learning by applying deep learning directly to raw waveforms, whereas in the past, machine learning was applied to features in the frequency domain. In the frequency domain, features that assume the stationarity of time series data for EEG tend to be detected. However, by applying end-to-end learning to the raw waveform, it may be possible to detect features that reflect non-stationary waveform features, such as those identified in clinical EEG, because the stationarity of the features is not assumed.

However, some previous studies have been validated on datasets with a small number of participants, for example, nine participants (Sakhavi et al., 2015, 2018; Schirrmeister et al., 2017; Lawhern et al., 2018). In such cases, there is a need to exercise caution, particularly when the performance validation is carried out within-subject, which is a method of learning with the same participant data as the test data. This method, however, does not guarantee high generalization performance to unknown participants, in which case calibration is not needed.

In the recent years, there have been validations with large datasets consisting of more than 50 participants, but generalizable models that do not require calibration have not been built, possibly for reasons such as the existence of participant groups with low accuracy (Lee et al., 2020) or the requirement for calibration concerning each participant (Lun et al., 2020). From the practical viewpoint of application of BCI, it requires consideration as to whether calibration should be actively carried out. For example, in a BCI such as a game device (Ahn et al., 2014; Kerous et al., 2018), which simply needs to move exactly as the participant desires, calibration using the participant's own EEG will not be problematic if it is acceptable to the participant. However, BCI is not only used in applications such as gaming devices for the purpose of entertainment, but also for long-term training purposes to improve users' abilities or to treat diseases.

When applied in a medical field such as rehabilitation, training with neurofeedback may be conducted using orthoses or other devices that are moved by BCIs to achieve motor function recovery (Shindo et al., 2011; Kasashima et al., 2012; Ramos-Murguialday et al., 2013; Ang et al., 2014; Frolov et al., 2017; Ibáñez et al., 2017; Biasiucci et al., 2018). In such a case, if calibration is to be carried out, it should be performed to improve the effect of treatment, to expand the number of participants to whom the BCI can be applied, and to obtain clinical significance (Ramos-Murguialday et al., 2013; Ono et al., 2014; Ibáñez et al., 2017).

However, in the case of models whose performance has been improved by within-subject cross-validation, it is required as a matter of policy that performance be guaranteed using only the data of the training participants in the calibration. Although BCIs for training purposes such as therapeutic use should be adjusted to be closer to the generalized model of the training target, this policy implies that the BCI is only closer to the supervised label presented to the training participants in the calibration session and the training participants' own brain activity data at that time, and not to the training target. In therapeutic scenarios, this implies that we are only approaching the patient's own model as a training participant, even though the training participants should be induced to be healthy persons for motor function recovery.

One of the reasons for the above policy is that most of the current studies are aimed at increasing the accuracy of within-subject cross-validation, increasing the accuracy in cross-subjects, and building a generalized model of the induction target of the training participants, which does not ensure the base accuracy without calibration. Training from the generalized model to the individual model by performing calibration for increasing the training effect of individual participants may contribute to the practical use of BCI, but if the performance of the generalized model is high initially, the need to rely on calibration will be reduced. Thus, BCI devices that can be used without calibration are generally focused only on the advantage of saving time and effort of preparation before use. However, because of the high robustness owing to the generalized model, they may also contribute to the induction effect and performance guarantee for use in neurofeedback for training purposes. Therefore, the basic policy of our research is to build a generalized model for the induction target of the training participants for BCI training, that is, to build a generalized model of healthy participants in BCI for therapeutic purposes.

Another important improvement for the development of BCI is that multilayer CNNs can be effective for improving the accuracy of BCI. In the recent studies, it has been recommended to insert residual networks into multilayer CNNs to solve the degradation problem of multilayer neural networks (Simonyan and Zisserman, 2014). Residual networks have the advantage of solving the degradation problem during training and contributing to accuracy improvement. The degradation problem is a phenomenon in which, because CNN is multilayered (for example, more than 20 layers in one previous study), it is challenging to improve the error on the training data rather than improving the error on the test data as the layers become deeper. According to research in image recognition, residual nets can solve the degradation problem by adding a shortcut connection, which is a copy of the input, after the CNN layer, to prevent performance degradation by inserting more layers. However, previous BCI studies have reported that the insertion of residual networks adversely affects classification accuracy (Schirrmeister et al., 2017).

In our study, we evaluate the effect of residual nets for BCI not by within-subject validation of previous studies, but by cross-subject validation using preprocessing, multilayering, and as much data as possible. We consider that there is still a possibility that the insertion of residual networks can contribute to the improvement of classification accuracy by improving CNN-based EEGNet (Lawhern et al., 2018), which can apply end-to-end learning on the raw EEG, and learning it with large datasets. After the batch normalization layer, we insert a residual network into the multilayered blocks and use an EEG dataset of more than 100 healthy participants obtained while they were resting, executing left-hand movement, and executing right-hand movement to validate the classification accuracy by cross-validation.

As scientific and medical findings indicate that event-related desynchronization (ERD), recorded near the sensorimotor area (SM1), is involved in hand movements (Pfurtscheller and Lopes da Silva, 1999), some conventional BCIs that classify hand movements from EEG are controlled by handcrafted features designed to capture only ERD (Shindo et al., 2011; Ang et al., 2014; Ono et al., 2014; Ibáñez et al., 2017). Even if they try to design BCIs using phenomenon and evaluation indices that are statistically significant using known neuroscience findings, they do not necessarily show high generalization performance, and it is probable that the neuroscience findings necessary for high-performance BCIs are not fully understood. By contrast, with the development of machine learning technology, there is room for the development of high-performance BCIs if large amounts of data are available. Furthermore, recently, technologies such as Grad-CAM (Selvaraju et al., 2017) that can visualize the reasons for CNN decisions have been developed, and it is now possible to obtain neuroscientific findings from high-performance BCIs with generalized models. That is, it is possible to obtain the type of neuroscientific findings required for generalized models by considering a different approach from the conventional findings.

There are two sides to this approach: (1) the previously known neuroscientific findings and (2) new findings, but with improved performance that will allow us to obtain new findings. However, the way in which the cross-validation was conducted should be noted. Previous studies evaluated within-subject cross-validation and visualized the model, so that it represented individual features of within-subject, but it could not describe generalized features across participants (Schirrmeister et al., 2017; Lawhern et al., 2018). Upon high-accuracy classification of whether the BCI proposed in this study executes the hand movements of healthy participants and demonstrating this using the test data of unknown participants against training data, we can visualize the model and obtain generalized knowledge across participants as to when and which brain region influences the hand movements of healthy participants.

This study was conducted to develop a generalized BCI model, visualize the model, and classify hand movements in healthy participants as an induction target for training, thereby demonstrating that the data-driven scheme of scientific exploration is valid and has neuroscientific value.

## Materials and Methods

This section describes the datasets used to train, validate, and test the proposed model, its architecture, and how to visualize the model.

### Dataset

A dataset with a large number of participants is required to tune the multilayer residual network and analyze the classification model on a test dataset with a sufficient number of participants. The PhysioNet EEG dataset (Goldberger et al., 2000; Schalk et al., 2004) was chosen; it consists of 109 participants from an open public EEG dataset during motor execution and imagery tasks. The analysis of the classification model was aimed at explaining the neurological process during movement in healthy participants. Thus, motor execution, which is less affected by participants' unfamiliarity and has higher label reliability compared with motor imagery, was focused upon, and the rest and motor execution data of both the right and left fists for the task were used, resulting in a three-task classification. The opening and closing fist movements were repeated. The duration of each task was 4.5 s.

The details of the EEG motor execution dataset from PhysioNet are as follows:

Participants: 109 healthy participants

Tasks: rest, left-hand fist, right-hand fist

Sampling frequency: 160 Hz

**Figure 1:**
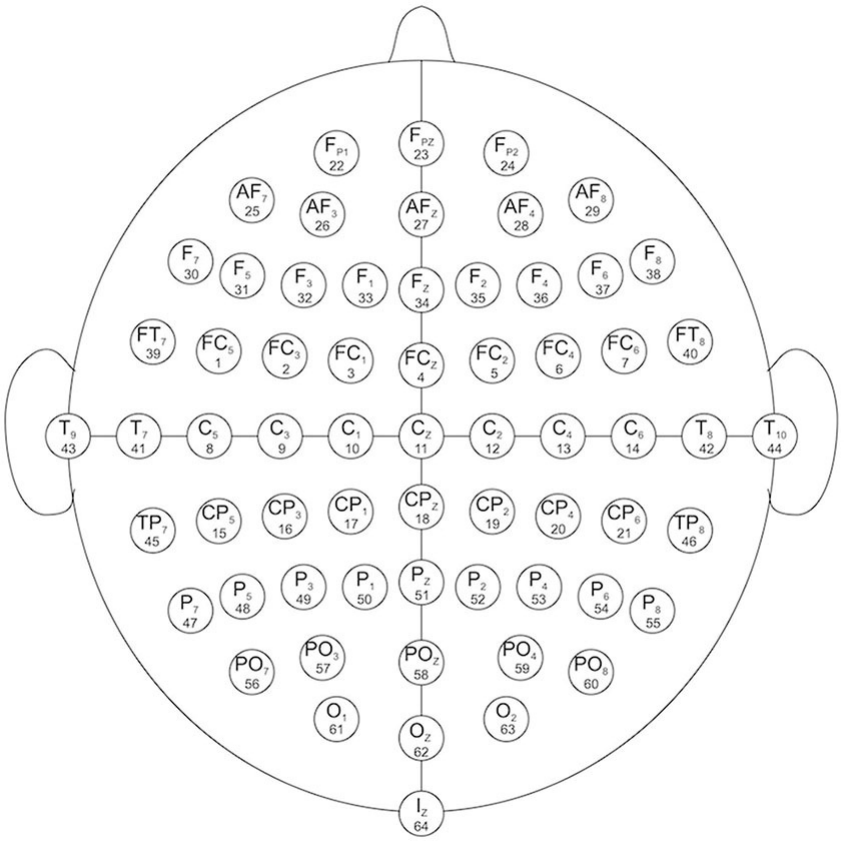
EEG channels map (Schalk et al., 2004).

EEG channels: 64 electrodes as, per the international 10–10 system (Figure 1)

Trials: 30 trials (one trial selects any task)

Sessions: three sessions

There are 109 participants × 3 sessions × 30 trials data.

To perform data expansion on the training data during the data preprocessing, the EEG was normalized to the mean value of 0, standard deviation of 1, and the noise generated by normalized random numbers with 10% standard deviation in each trial scale and 3% in each channel scale were added. Because four participants (#88, #89, #92, and #100) did not complete the experiment, their data were not used in the study.

The data of 20 participants out of 105 were randomly assigned as test data, from which the data of the four abovementioned participants were removed. Then, 5-fold cross-validation was performed on the data of the remaining 85 participants, and the model was evaluated by averaging the accuracy of the test data for 20 participants. Because the training, validation, and test data were divided by cross-subjects and not within-subjects, the data for the participants in the training, validation, and test were not mixed and did not overlap. In addition, when calculating the accuracy of the test and validation data, no calibration was conducted using data from the same participant.

### Model Architecture

**Figure 2:**
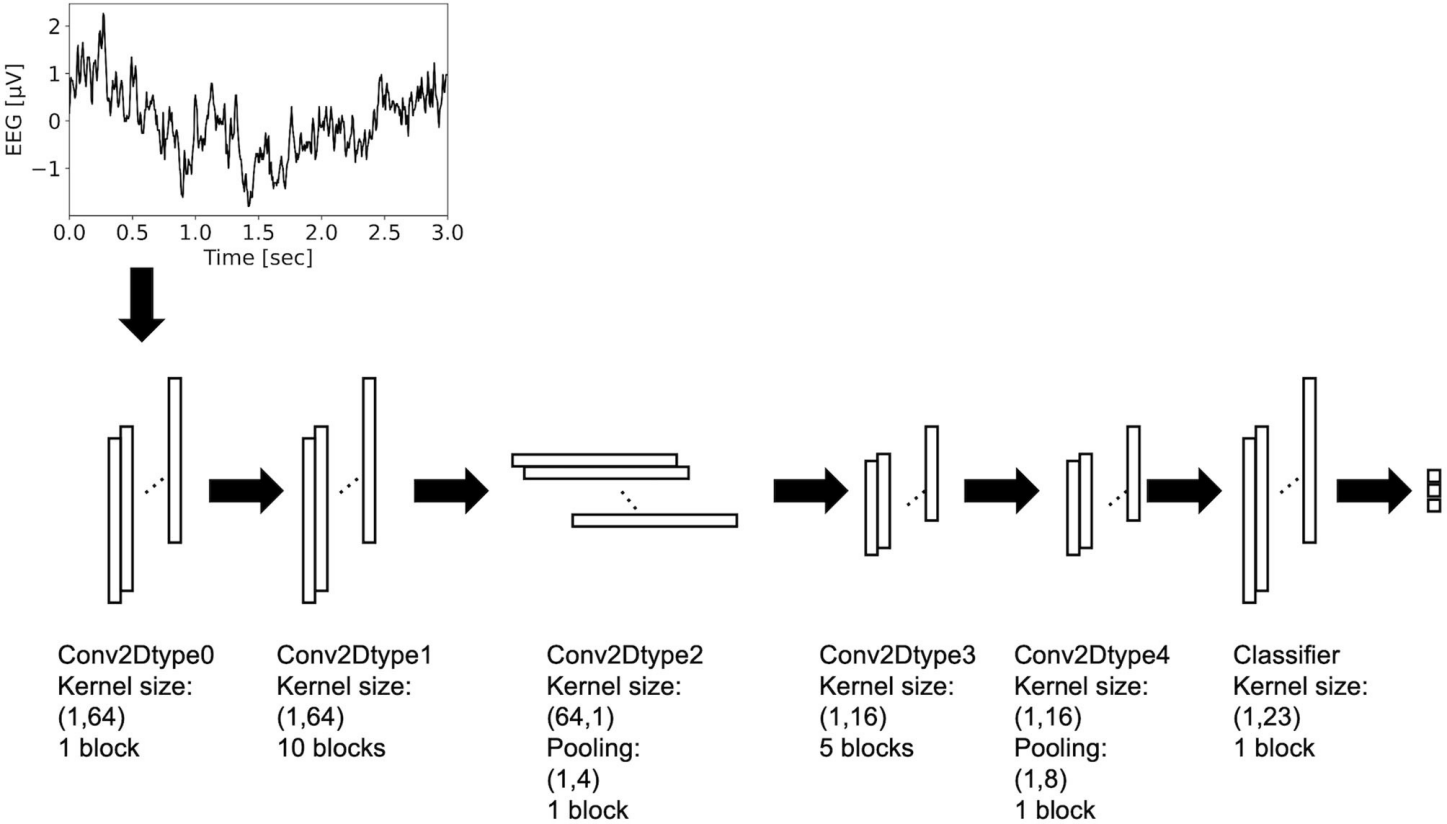
Architecture of residual-EEGNet. This model consists of Conv2Dtype0~4 blocks and classifier.

Residual-EEGNet (Figure 2) was constructed by revising EEGNet—a model from a previous study (Lawhern et al., 2018). The following is a description of the blocks used in its architecture. The variable F1 was set to eight, D was set to two, F2 was set to 16, and N_class was set to three.

The Conv2Dtype0 block consists of a convolution layer (Krizhevsky et al., 2012) and a batch normalization layer (Ioffe and Szegedy, 2015) in that order. The convolution layer performs convolution with a kernel size of (1, 64) for a compressed 2D vector (channels scale, time scale). The number of input feature maps is one and the number of output feature maps is F1.

**Figure 3:**
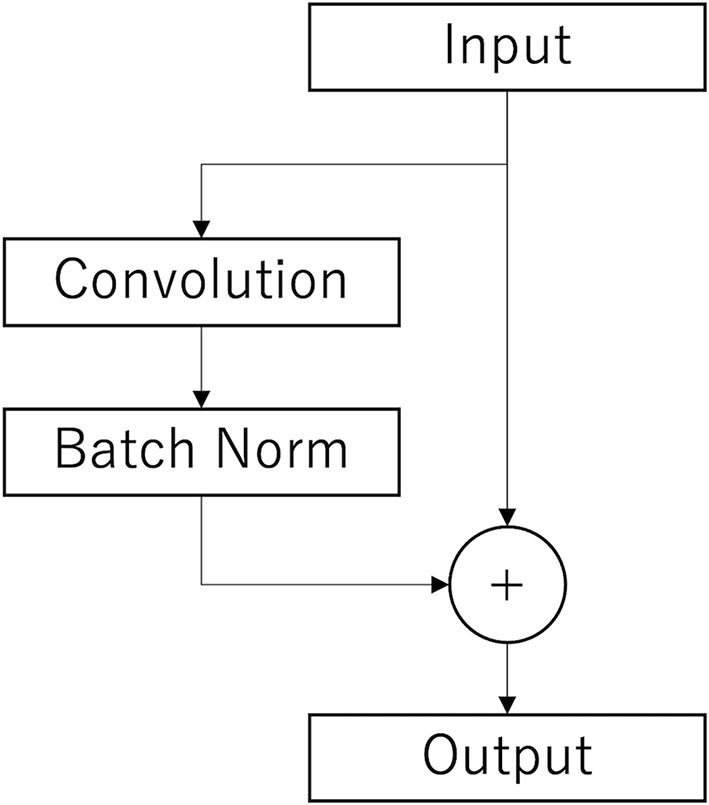
Architecture of the residual layer. After the convolutional layer and batch n normalization layer (Batch Norm), copies of the inputs are added to those outputs. This is the final output.

The Conv2Dtype1 block consists of a convolution layer, a batch normalization layer, and a residual block (Simonyan and Zisserman, 2014) in order (Figure 3). The convolution layer performs convolution with a kernel size of (1, 64) for a compressed 2D vector (channel scale, time scale). The number of input feature maps is F1 and the number of output feature maps is F1.

The Conv2Dtype2 block consists of a convolution layer, a batch normalization layer with an exponential linear unit (ELU) activation function, average pooling layer, and dropout layer (Krizhevsky et al., 2012), in that order. The convolution layer performs convolution with a kernel size of (64, 1) for a compressed 2D vector (channel scale, time scale). The convolution filter is regularized using a maximum norm constraint of one on its weights. The average pooling layer performs convolution with a kernel size of (1, 4) for a compressed 2D vector (channel scale, time scale). The dropout ratio is 25%. The number of input feature maps is F1 and the number of output feature maps is F1 × D.

In the Conv2Dtype3 block, two convolution layers for depthwise convolution and pointwise convolution (Chollet, 2017) are used, as in the EEGNet. The depthwise convolution layer has a convolution with a kernel size of (1, 16) for a compressed 2D vector (channel scale, time scale). The number of input feature maps is F1 × D and the number of output feature maps is F1 × D. The pointwise convolution layer has a convolution with kernel size of (1, 1), the number of input feature maps is F1 × D, and the number of output feature maps is F1 × D. After depthwise convolution and pointwise convolution for the input, Conv2Dtype3 has a batch normalization layer, and a residual block, in that order (Figure 3).

In the Conv2Dtype4 block, two convolution layers for depthwise convolution and pointwise convolution are used, as in EEGNet. The depthwise convolution layer has a kernel size of (1, 16) for a compressed 2D vector (channel scale, time scale). The number of input feature maps is F1 × D, and the number of output feature maps is F1 × D. The pointwise convolution has a kernel size of (1, 1), the number of input feature maps is F2, and the number of output feature maps is F1 × D. After depthwise convolution and pointwise convolution for the input, Conv2Dtype4 contains a batch normalization layer, residual block with the ELU activation function (Clevert et al., 2016), average pooling layer, and dropout layer. The average pooling layer performs convolution with a kernel size of (1, 8) for a compressed 2D vector (channel scale, time scale). The dropout ratio is 25%.

In the Classifier block, the input is processed and the output is processed through a convolution layer with a kernel size of (1, 23) using the LogSoftmax function. The number of input feature maps is F2 and the number of output feature maps is N_class units, with N_class being the number of classes in the data.

The architecture presented above is that of Residual-EEGNet. Residual-EEGNet is coupled with a forward flow of one block of Conv2Dtype0, ten blocks of Conv2Dtype1, one block of Conv2Dtype2, five blocks of Conv2Dtype3, one block of Conv2Dtype4, and one Classifier block (Figure 2). Non-Residual-EEGNet has the same architecture, but the residual block present in Residual-EEGNet is absent; Non-BN-EEGNet has the same architecture but the batch normalization layer is absent; Non-Dropout-EEGNet has the same architecture, but the dropout layer is absent. Non-Preprocessing is the method without the data preprocessing from Residual-EEGNet. EEGNet, Deep-ConvNet, and Shallow-ConvNet are legacy models that were used in the previous studies (Schirrmeister et al., 2017; Lawhern et al., 2018).

Stochastic gradient descent (SGD) (Robbins and Monro, 1951; Rumelhart et al., 1986; Zhang, 2004; Bottou, 2010; Bottou et al., 2018) was employed to optimize the model parameters with cosine annealing of the learning rate (Loshchilov and Hutter, 2017). The categorical cross-entropy loss function was minimized. A total of one thousand training iterations (epochs) were run. The initial learning rate was set to 0.001 and momentum to 0.9 in the SGD optimizer, and the maximum update period was set to 29,000 iterations in cosine annealing. The batch size was set to 100. These three network models were optimized using a training dataset.

### Visualization Method of the BCI Classification Process

Class activation mapping (CAM) (Zhou et al., 2016)-based approaches are often used to provide a visual understanding of predictions made by CNNs. Because the CAM algorithm assumes a neural network with a global average pooling (GAP) layer (Lin et al., 2013), which calculates the average value for each feature map and assigns that value to the subsequent neural network, we used Grad-CAM (Selvaraju et al., 2017), which is generalized to allow visualization of the reasons for classification in CNN architectures without GAP.

In CAM, the weights connecting the GAP calculation values and the output layer of the neural network are applied to the feature map to visualize the regions in the image that are used as decision criteria.

CAM: For the CNNs containing GAP layers, which this algorithm can support, the weighted sum of the GAP values determines the final output class of the image slice.

Plugging $F_{k}=\underset{x,y}{\sum }f_{k}\left(x,y\right)$ into the class score, *S*_*c*_, yields


$$\begin{array}{l} S_{c}=\underset{k}{\sum }w_{k}^{c}\underset{x,y}{\sum }f_{k}\left(x,y\right)=\underset{x,y}{\sum }\underset{k}{\sum }w_{k}^{c}f_{k}\left(x,y\right). \end{array}$$

We define *M*_*c*_ as the class activation map for class *c*, where each spatial element is given by


$$\begin{array}{l} M_{c}\left(x,y\right)=\underset{k}{\sum }w_{k}^{c}f_{k}\left(x,y\right). \end{array}$$

Thus, $S_{c}=\underset{x,y}{\sum }M_{c}\left(x,y\right)$, and hence, *M*_*c*_(*x, y*) directly indicates the importance of the activation at spatial grid (*x, y*), leading to classification of an image as class *c* (Zhou et al., 2016). This computation maps the importance of the information from the output, which is used to classify decisions, directly into each layer.

Grad-CAM: We differentiate the probability score *y*^*c*^ of class *c* with respect to the intensity $A_{ij}^{k}$ at the (*i, j*) pixel of the *k*th feature map and calculate the gradient $\frac{\partial y^{c}}{\partial A_{ij}^{k}}$. By averaging them over all pixels (global average pooling), we calculate the weight factor $\alpha_{k}^{c}$ for the *k*th filter of class *c*,


$$\begin{array}{l} \alpha_{k}^{c}=\frac{1}{z}\sum_{i}\sum_{j}\frac{\partial y^{c}}{\partial \;A_{ij}^{k}}. \end{array}$$

The larger the value of weight factor $\alpha_{k}^{c}$, the more influence the feature map *A*^*k*^ has on class *c*.

Consequently, the heatmap generation used the following formula:


$$\begin{array}{l} L_{Grad-CAM}^{c}=ReLU\;\left(\sum_{k}\alpha_{k}^{c}A^{k}\;\right), \end{array}$$

where the rectified linear unit (ReLU) function was only allowed to evaluate features with positive impact. The weighted average of the *k* filters is calculated using weighting factor $\alpha_{k}^{c}$, and the output is calculated using activation function *RELU* (*x*) ≡ *max*{*x*, 0}, defined as the heatmap output.

Thus, Grad-CAM can visualize the reasons for decisions using the gradient between the feature map and the output values of the neural network. This study employed Grad-CAM as a visualization method for the BCI classification process. Here, the convolutional layer of the block, which is one block before the dimension reduction block in the first pooling layer, was used for visualization, although it is said that visualizing the last convolutional layer can reveal the most discriminative part locally based on the results of Grad-CAM analysis.

## Results

### Comparison With the Cross-Validation of Cross-Subject in Terms of BCI Performance

**Figure 4:**
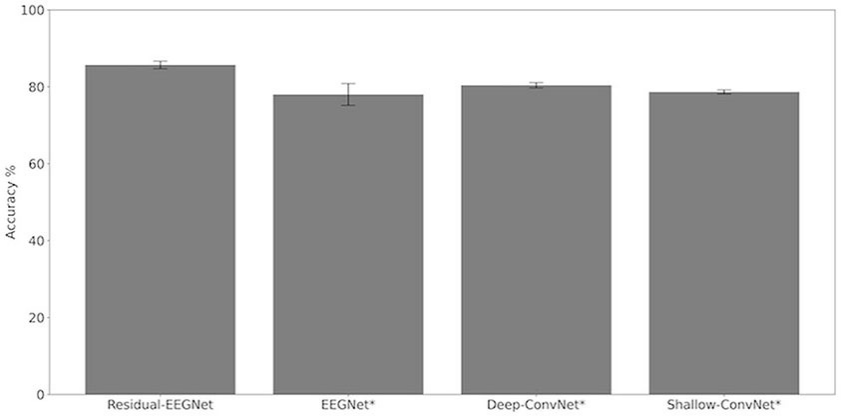
Comparison of decoding accuracy for four models. The mean and standard deviation of the accuracy in the test data were evaluated by cross-validation. The error bar shows the standard deviation. The accuracy of Residual-EEGNet was higher than that of EEGNet, Deep-ConvNet, and Shallow-ConvNet and was statistically significant (*p < 0.05).

**Figure 5:**
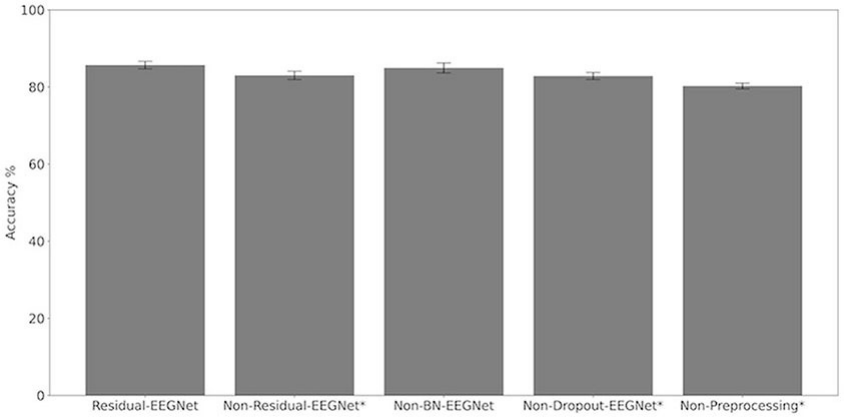
Comparison of decoding accuracy for five models. The mean and standard deviation of the accuracy in the test data were evaluated by cross-validation. The error bar shows the standard deviation. The accuracy of Residual-EEGNet was higher than that of Non-Residual-EEGNet, Non-BN-EEGNet, Non-Dropout-EEGNet, Non-Preprocessing and was statistically significant (*p < 0.05).

Figures 4, 5 and Table 1 compare the accuracies of Residual-EEGNet (the proposed model), Non-Residual-EEGNet (the model without a residual network), Non-BN-EEGNet (the model without a batch normalization layer), Non-Dropout-EEGNet (the model without a dropout layer), Non-Preprocessing (the model without data preprocessing), EEGNet (a legacy model), Deep-ConvNet, and Shallow-ConvNet. Here, statistical comparisons were made using Welch's *t*-test (Welch, 1938, 1947) for each of the two unpaired samples, and the applied threshold was set to control the false discovery rate (FDR) at *p*-value using the Benjamini–Hochberg procedure (Benjamini and Hochberg, 1995). The Benjamini–Hochberg procedure prevents type I errors. On comparing the decoding accuracy of Residual-EEGNet to Non-Residual-EEGNet, Non-BN-EEGNet, Non-Dropout-EEGNet, and Non-Preprocessing for the ablation study, three statistically significant differences without Non-BN-EEGNet (Figure 4) were observed. On comparing the decoding accuracy of Residual-EEGNet to EEGNet, Deep-ConvNet, and Shallow-ConvNet, which are legacy models, three statistically significant differences (*p* < 0.05) were observed (Figure 5).

**Table 1:** Differences in the decoding accuracies of Residual-EEGNet, Non-Residual-EEGNet, Non-BN-EEGNet, Non-Dropout-EEGNet, Non-Preprocessing, EEGNet, Deep-ConvNet, and Shallow-ConvNet.

	**Residual-EEGNet**	**Non-Residual-EEGNet**	**Non-BN-EEGNet**	**Non-Dropout-EEGNet**	**Non-Preprocessing**	**EEGNet**	**Deep-ConvNet**	**Shallow-ConvNet**
Mean %	85.69	83.00	84.95	82.84	80.25	78.01	80.40	78.66
Std %	1.10	1.22	1.45	1.03	0.83	3.18	0.780	0.585
*P*-value (diff to Residual-EEGNet)	–	0.0064^*^	0.39	0.0029^*^	0.000033^*^	0.0039^*^	0.000042^*^	0.000013^*^

*The accuracy of Residual-EEGNet was higher than those of Non-Residual-EEGNet, Non-Dropout-EEGNet, Non-Preprocessing, EEGNet, Deep-ConvNet, and Shallow-ConvNet, with statistical significance (*

* *p < 0.05)*.

### Grad-CAM Results

**Figure 6:**
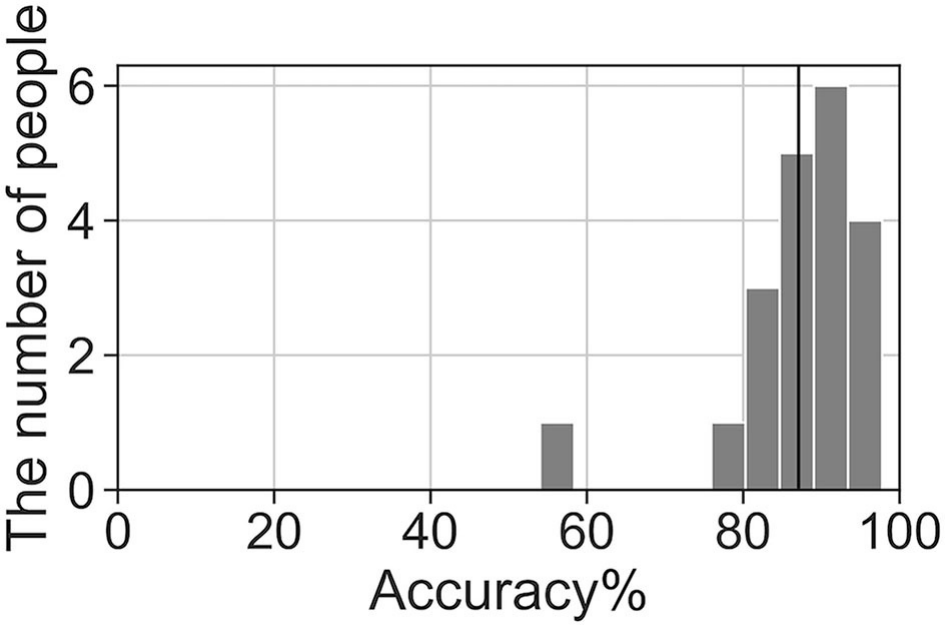
Histogram of accuracy averaged by participants for the highest accuracy Residual-EEGNet model. The black line represents the total average accuracy (87.1%).

We analyzed the accuracy of the brain activity classification results obtained using our BCI model. For the visualized model, the highest accuracy (87.1%) of the Residual-EEGNet model for cross-validation was selected. The confusion matrix of the model is shown in Table 2. The histogram of the accuracy averaged for participants in the model is shown in Figure 6. Except for one participant, the results exhibit high accuracy. For the data classified by the selected model, the classification was visualized using Grad-CAM to obtain the correct answers where the actual label and the estimated value matched. For the selected layer for Grad-CAM, the 10th layer was chosen, the last of the Conv2Dtype1 block, to locally analyze the parts that contributed more to the classification. The 10th layer was a 2D layer of features for 64 channels and 720 time points.

**Table 2:** Confusion matrix for the highest accuracy (87.1%) residual-EEGNet model.

		**Predicted**		
		**Rest**	**Left**	**Right**
Actual	Rest	810	38	52
	Left	40	361	27
	Right	39	31	357

**Figure 7:**
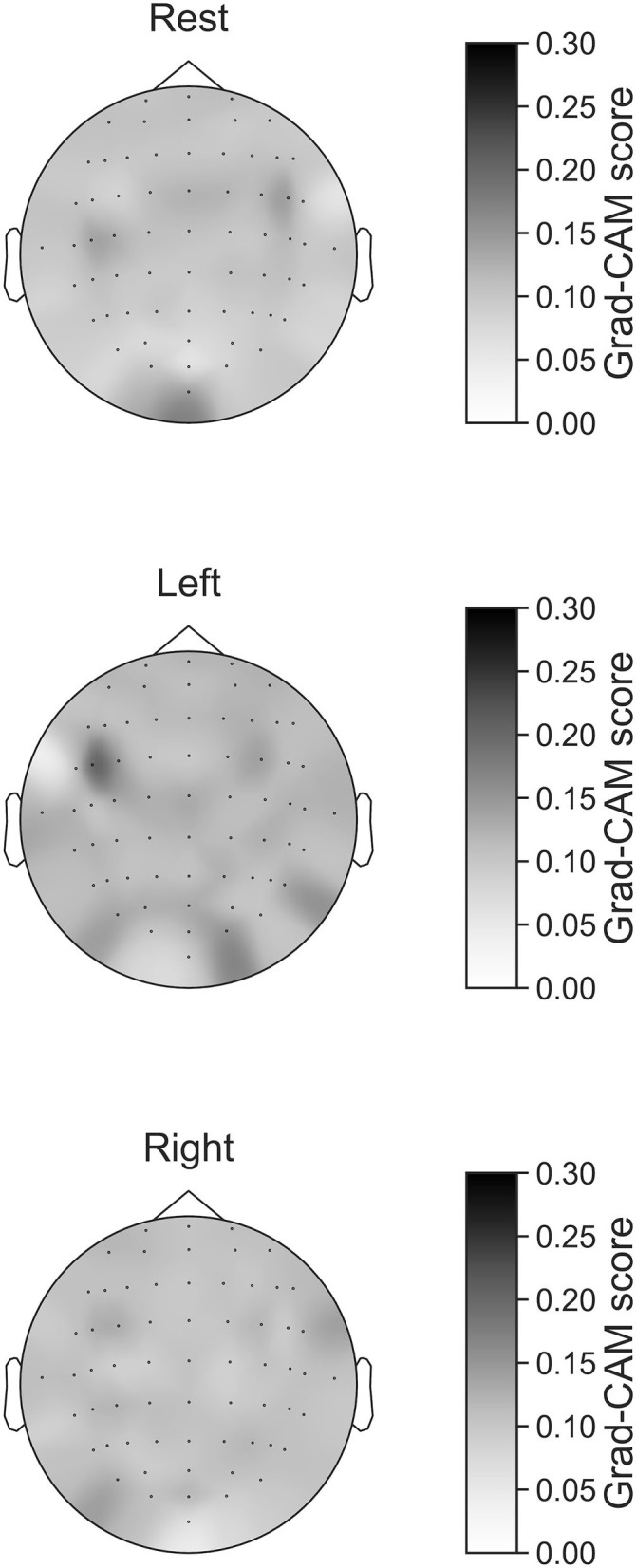
Heatmap of Grad-CAM score on Residual-EEGNet for three tasks (rest, left-hand motor execution, and right-hand motor execution). The heatmap was made from Grad-CAM scores of 64 channels.

**Figure 8:**
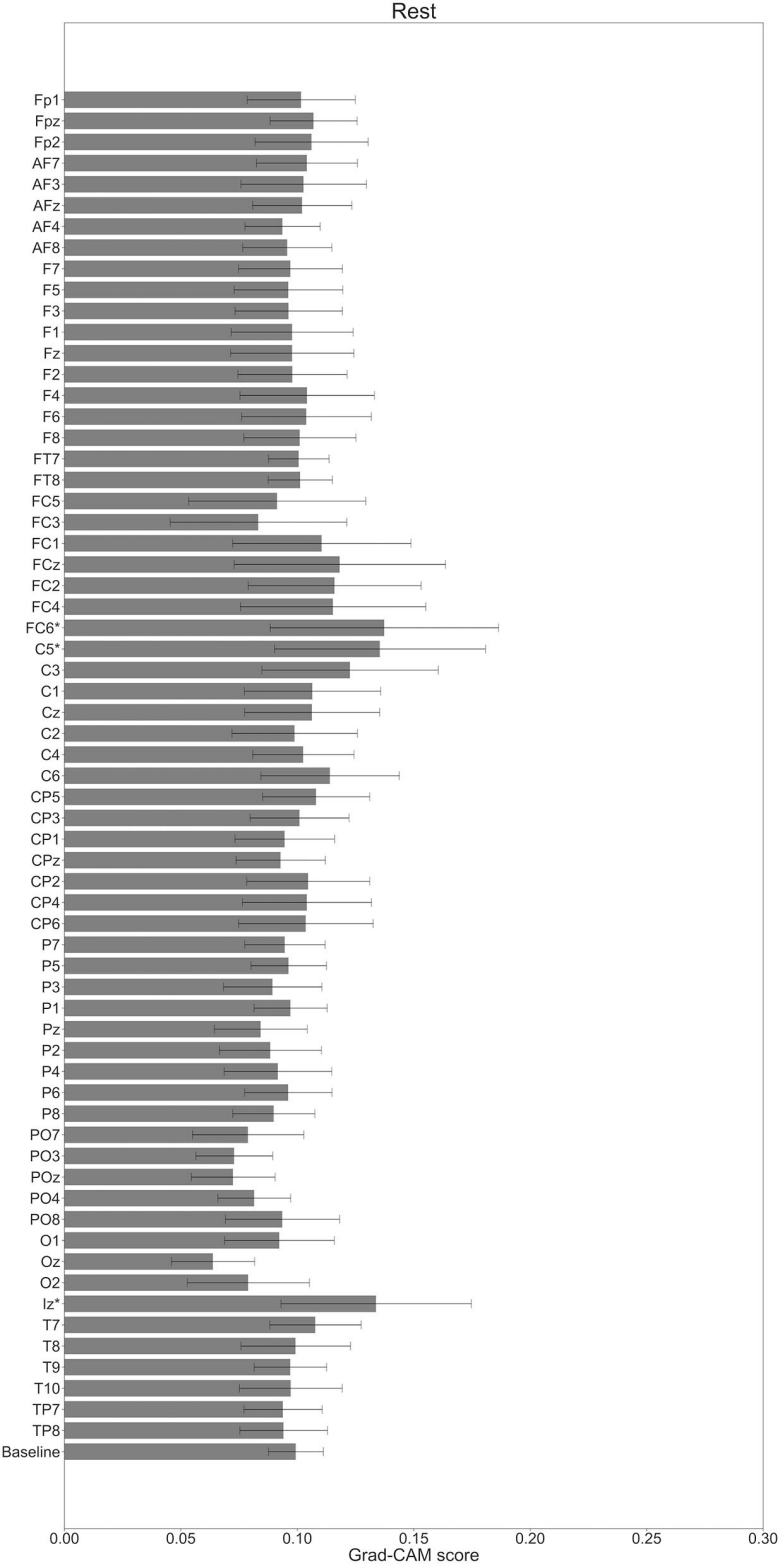
Comparison of all channels' Grad-CAM scores on Residual-EEGNet for rest. The baseline was calculated as the mean of 64 channels' scores. The Grad-CAM scores for FC6, C5, and Iz were higher than the baseline, and were statistically significant (*p < 0.05). The error bar shows the standard deviation between participants.

**Figure 9:**
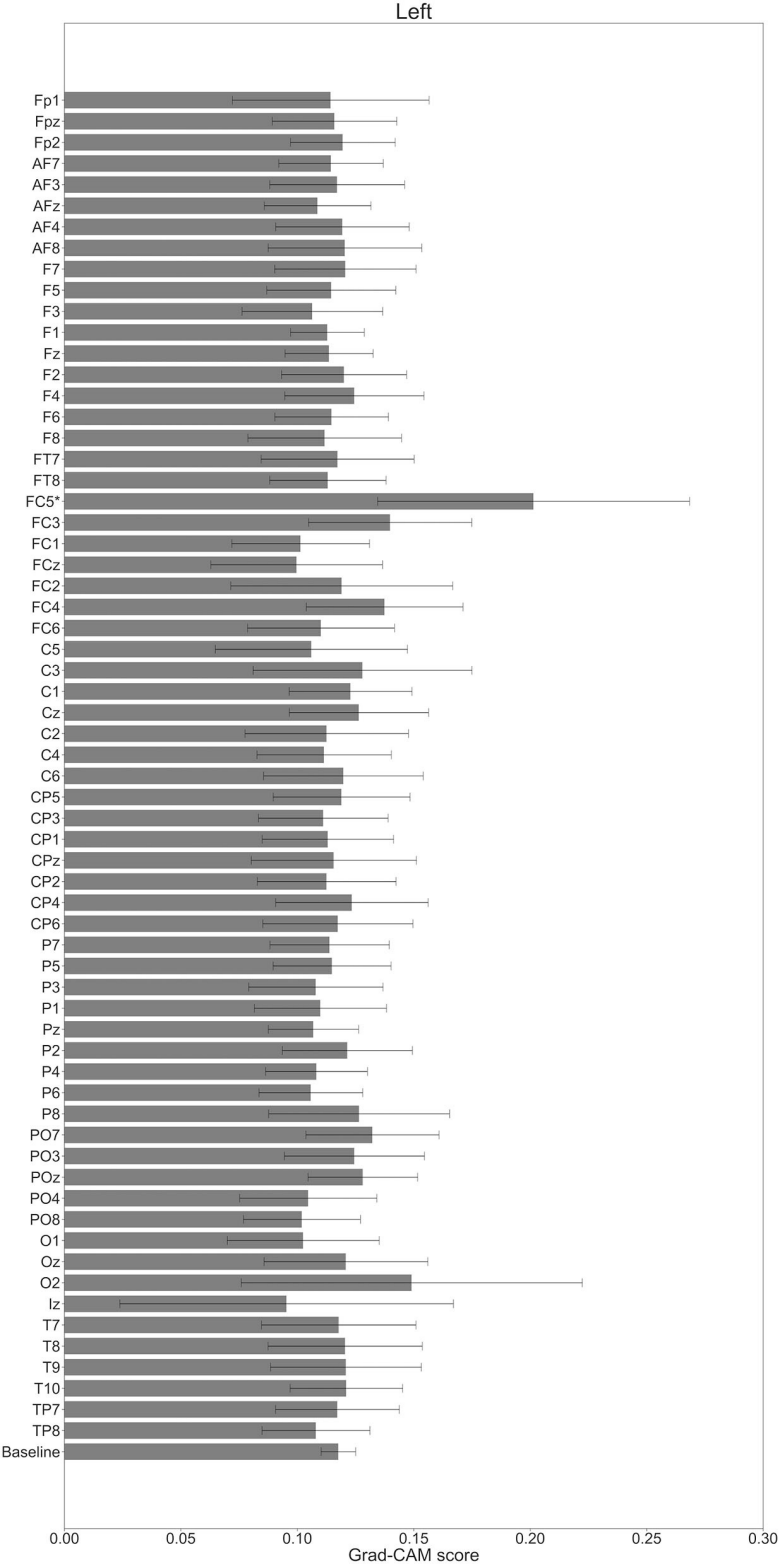
Comparison of all channels' Grad-CAM scores on Residual-EEGNet for left-hand motor execution. The baseline was calculated as the mean of 64 channels' scores. The Grad-CAM score for FC5 was higher than the baseline and was statistically significant (*p < 0.05). The error bar shows the standard deviation between participants.

**Figure 10:**
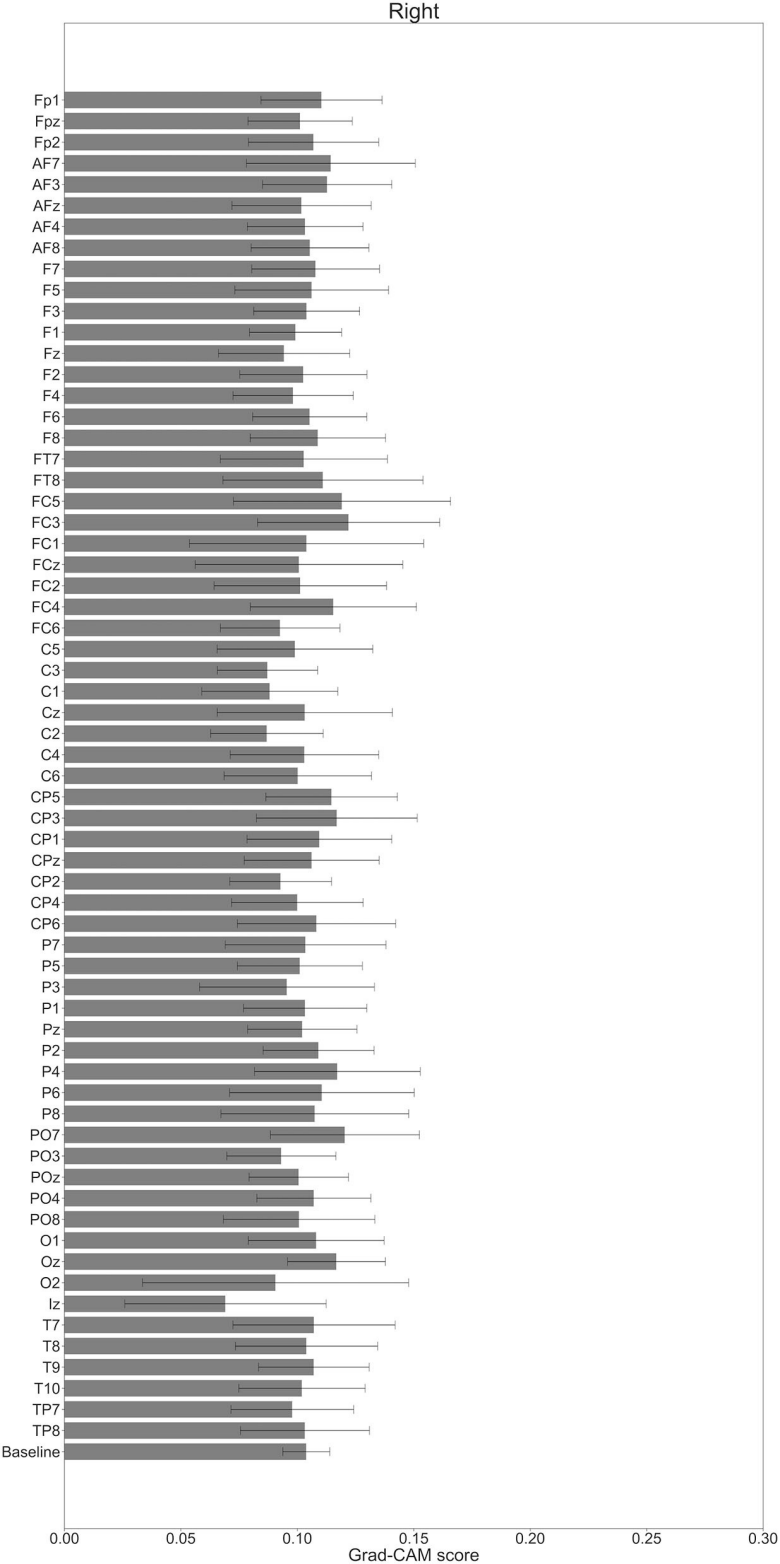
Comparison of all channels' Grad-CAM scores on Residual-EEGNet for right-hand motor execution. The baseline was calculated as the mean of 64 channels' scores. No Grad-CAM score was statistically significant (*p < 0.05). The error bar shows the standard deviation between participants.

In Figures 7–10, the mean in the time domain of the Grad-CAM scores is shown for the three tasks (rest, left-hand motor execution, and right-hand motor execution) on Residual-EEGNet. In Figure 7, the mean Grad-CAM scores are shown in the heatmap. In Figures 8–10, we show the mean of the Grad-CAM scores and the standard deviation of the participants. The baseline is also shown—obtained by calculating the average of all the channels. In Figures 8, 10, the channels with locally high scores were identified by statistically comparing the difference between each channel's Grad-CAM score and the baseline. Here, statistical comparisons were made using Welch's *t*-test (Welch, 1938, 1947) for each of the two unpaired samples, where the applied threshold should be set to control the FDR at *p*-value by the Benjamini–Hochberg procedure (Benjamini and Hochberg, 1995). For the rest task, Grad-CAM scores of FC6, C5, and Iz were locally higher than the baseline, with statistical significance (^*^p < 0.05). For the left-hand motor execution task, the Grad-CAM score of FC5 was locally higher than the baseline, with statistical significance (^*^p < 0.05). These localized increases in Grad-CAM score are also confirmed in Figure 7. On the other hand, for the right-hand motor execution task, no channel was locally higher than the baseline statistically.

**Figure 11:**
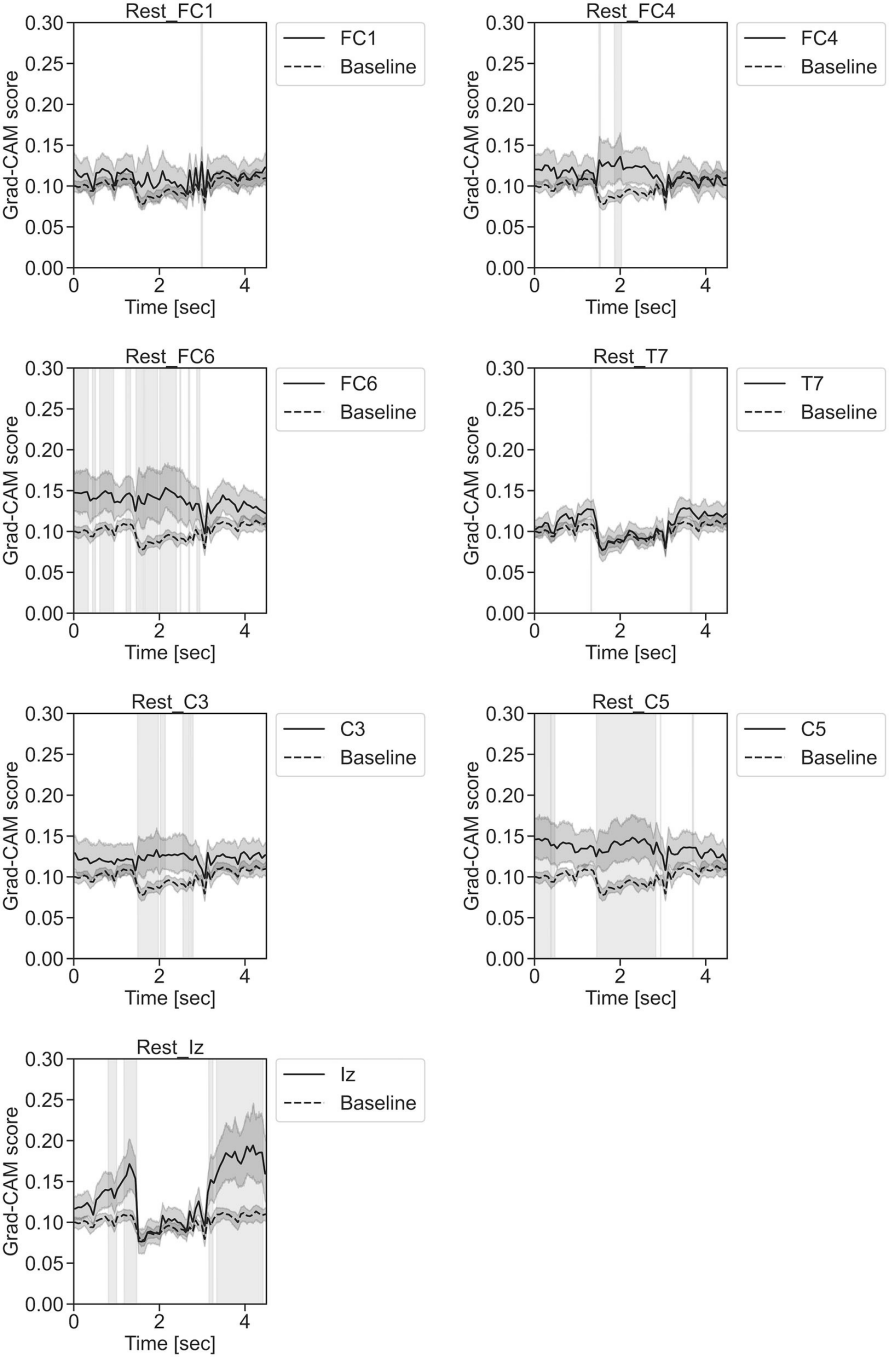
Time series changes of Grad-CAM scores during rest. In the gray-filled background, the Grad-CAM scores for the FC1, FC4, FC6, C3, C5, Iz, and T7 channels were higher than the baseline and were statistically significant (*p < 0.05).

**Figure 12:**
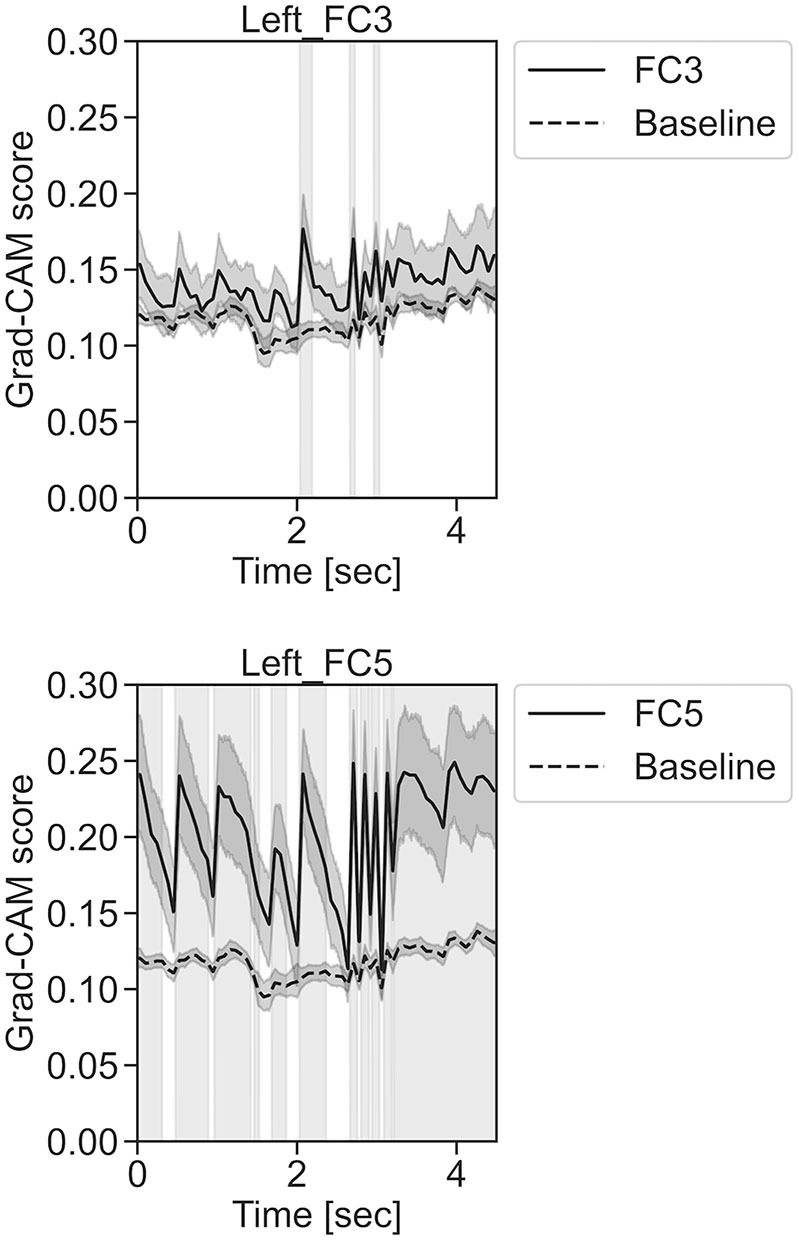
Time series changes of Grad-CAM scores during left-hand motor execution. In the gray-filled background, the Grad-CAM scores for the FC3 and FC5 channels were higher than the baseline and were statistically significant (*p < 0.05).

**Figure 13:**
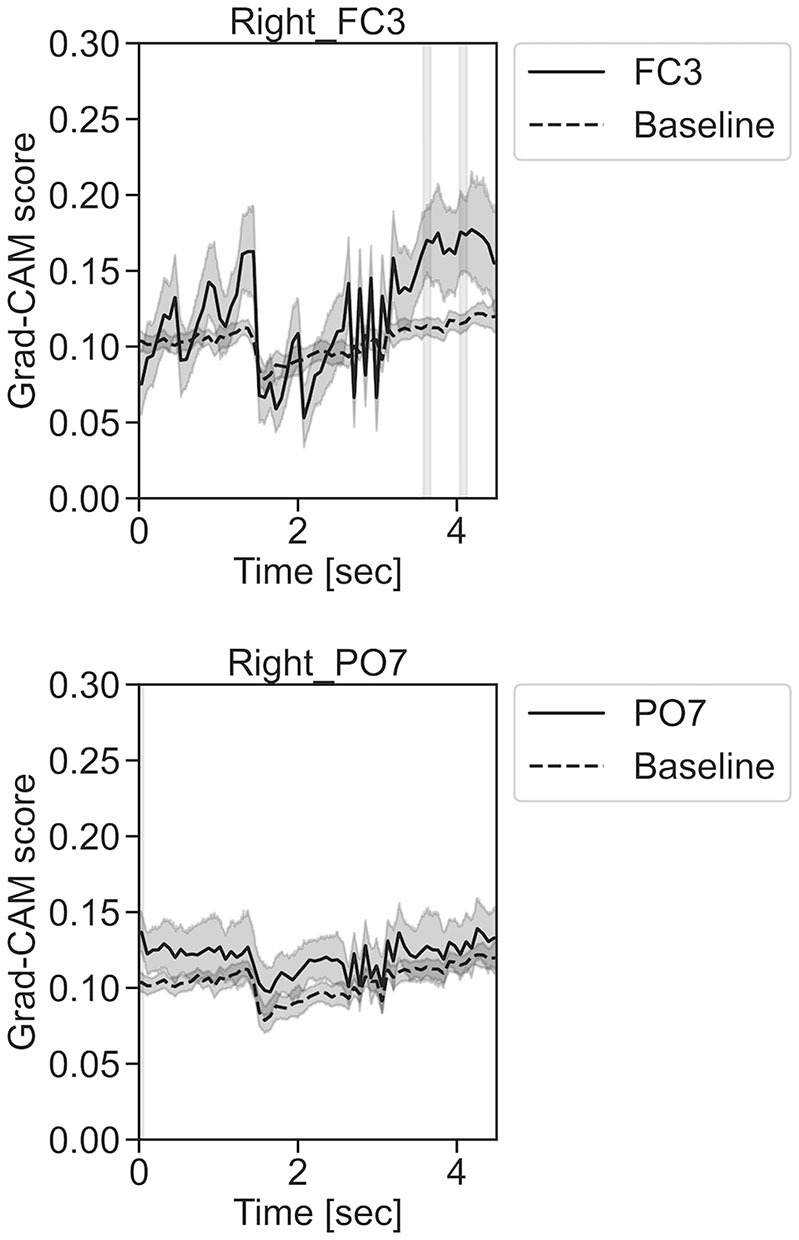
Time series changes of Grad-CAM scores during right-hand motor execution. In the gray-filled background, the Grad-CAM scores for the FC3 and PO7 channels were higher than the baseline and were statistically significant (*p < 0.05).

In Figures 11–**13**, the changes in the Grad-CAM scores in the time domain for each channel are shown. By statistically comparing the difference between each channel's Grad-CAM score and the baseline for each time point, we identified the time zones with locally high scores for each channel. Similarly, statistical comparisons were made using Welch's *t*-test (Welch, 1938, 1947) for each of the two unpaired samples, where the applied threshold should be set to control the FDR at *p*-value using the Benjamini–Hochberg procedure (Benjamini and Hochberg, 1995).

In Figure 11, for the rest task, only Grad-CAM scores in the FC1, FC4, FC6, C3, C5, Iz, and T7 channels were locally higher than the baseline at specific time points and were statistically significant (^*^p < 0.05). The scores of the other channels did not significantly increase. In particular, the Grad-CAM scores in FC6, C3, C5, and Iz significantly increased for an extended period. In Figure 12, regarding the left-hand motor execution task, only the Grad-CAM scores in the FC3 and FC5 channels were locally higher than the baseline at specific time points and were statistically significant (^*^p < 0.05). Meanwhile, the scores of the other channels did not significantly increase. In particular, the Grad-CAM scores in FC5 significantly increased for an extended period. In Figure 13, for the right-hand motor execution task, only the Grad-CAM scores in the FC3 and PO7 channels were locally higher than the baseline at specific time points and were statistically significant (^*^p < 0.05). The scores for the other channels did not significantly increase.

## Discussion

### BCI Performance Evaluation

The accuracy of Residual-EEGNet was significantly higher than that of Non-Residual-EEGNet (^*^p < 0.05). This implies that the residual network contributed to improving the accuracy. Schirrmeister et al. (2017) previously reported that introducing a residual network negatively affects the accuracy improvement and contributes to a decrease in the BCI accuracy, which classifies motor imagery with EEG. Unlike previous studies, which did not succeed in improving the accuracy by introducing residual networks, the current model achieved success, which is attributed to a significant difference compared to previous studies. Specifically, 85 subjects participated in 5-fold cross-validation and ~80% in training; by contrast, previous studies used datasets with fewer than 10 participants, for example, nine participants (Schirrmeister et al., 2017).

For reference, the ablation studies examined the performance of the model components by, respectively, removing two components (batch normalization and dropout) and the data preprocessing function from Residual-EEGNet. The ablation results for the two components, which are considered important components of Residual-EEGNet, showed that the accuracy of Non-Dropout-EEGNet was significantly lower than that of Residual-EEGNet. Batch normalization and dropout are the well-known methods for improving the accuracy of CNNs, and they are also used in the legacy EEGNet model, so removing them generally tends to reduce accuracy. However, the standalone removal of batch normalization did not significantly reduce the accuracy, and we consider that dropout, which significantly reduced the accuracy, contributed to the improved accuracy of Residual-EEGNet. The accuracy was also significantly lower when the data preprocessing function was removed. In this case, the removal of the data preprocessing function resulted in the raw waveforms being fed directly into the model, which may have affected the differences in impedance and noise conditions between subjects and between electrodes. The data preprocessing function in this study was found to be beneficial.

Furthermore, the accuracy of Residual-EEGNet was higher than that of EEGNet, Deep-ConvNet, and Shallow-ConvNet, and was statistically significant (^*^p < 0.05). As EEGNet was originally validated on a small dataset (nine participants) in the previous study, the accuracy of the model when trained with the different dataset under the different conditions was unknown (Lawhern et al., 2018). This study calculated the accuracy of the previous EEGNet when trained on the same dataset of over 100 participants as our Residual-EEGNet and compared it to the BCI model, confirming the improvement in accuracy over the previous EEGNet. In this comparison, the only difference between the current Residual-EEGNet and the previous EEGNet was the architecture of the model. The previous EEGNet (Lawhern et al., 2018) had three convolutional layers, whereas the current model has 10 layers, with a residual network inserted. This implies that the multiple layers of the residual CNNs improved accuracy.

From these results, a generalized model of motor classification for healthy people can be obtained by training the end-to-end learning model using raw EEG data. We found that inserting residual networks and multilayer networks improved the BCI accuracy and contributed to the development of a generalized model.

### Visualizing the Location and Timing of Neural Activities That Contribute to BCI Classification

The BCI model has a higher accuracy (87.1%) than previous models, such as EEGNet, Deep-ConvNet, Shallow-ConvNet (Schirrmeister et al., 2017; Lawhern et al., 2018). Consequently, the part of the brain activity that has a more generalized finding can be determined. The histogram of the accuracy averaged by participants for the model demonstrates that a generalizable model was successfully built. If the model is analyzed with Grad-CAM, generalizable knowledge about the brain processes related to hand movement can be obtained. In addition, because this study developed a more generalizable model for healthy participants, the model can be treated as an induction model of BCI for training.

The brain areas contributing to each task classification can be discussed based on the results of Grad-CAM. Regarding the rest task, a significant increase in Grad-CAM scores in the FC6, C3, C5, and Iz channels was observed. Notably, this suggests that these channels are related to specific characteristic cortical areas of rest against left- and right-hand motor execution, rather than all characteristics of rest. Furthermore, these channels reflect the activity of the premotor cortex (PMc), M1, and visual cortex (Kandel et al., 2000). In addition, a study on the development of BCI using supplementary motor area (SMA) and M1 as features trained within-subject has been published (Wang et al., 2006). Although not a generalized finding, Frolov et al. (2020) analyzed the classification component of BCIs for motor imagery trained using within-subject cross-validation and reported the involvement of SMA. Herein, it was assumed that activity near the PMc and SMA (Kandel et al., 2000), which are involved in motor execution planning, would become an apparent factor in classifying between rest and left- or right-hand motor execution, but the results of the current analysis did not show any brain activity near the SMA. In this regard, the results of this analysis only suggest that the PMc is at least highly localized and involved in classification and does not deny the involvement of activity other than that of the PMc in classifying between rest and motor execution.

In the field of image recognition, the results of Grad-CAM analysis of the final convolutional layer often show strong localization, but the results of this model generally show weak localization and non-zero baseline behavior. We consider that we have achieved high BCI accuracy by considering weak effects from various brain activity areas.

For the left-hand motor execution task, a significant increase in Grad-CAM scores in the FC3 and FC5 channels was confirmed. Notably, this suggests that these channels are related to specific characteristic brain sites of the left-hand motor execution task against rest and right-hand motor execution. However, for the right-hand motor execution task, a significant increase in the Grad-CAM scores in the FC3 and PO7 channels was observed. Notably, this suggests that the channels are related to specific characteristic brain sites of the left-hand motor execution task against rest and right-hand motor execution. These channels are thought to reflect the activity near the PMc.

The presence or absence of motor commands for the right- or left-hand can be classified in relation to the resting state using the activity near the SMA and PMc, called non-primary motor-related areas. However, it is not sufficient to discriminate between right- and left-hand motor commands, where it was assumed that it would be difficult to improve the accuracy without using the activity near M1, which has somatotopic localization. Anatomically, motoneurons in the right-hand are activated by motor commands from the M1 of the contralateral left hemisphere *via* the corticospinal tract, and *vice versa* (Kandel et al., 2000). Therefore, when using brain activity in the M1 to discriminate between left- and right-hand motor executions, it would be effective to discriminate between contralateral changes in brain activity in the right and left hemispheres, such as C4 and C3 on the electrode, respectively. However, the results of the Grad-CAM analysis failed to show any contrasting localization of the brain activity changes in C4 and C3, respectively, but showed a biased localization near the PMc of the left hemisphere, such as FC3 and FC5. FC3 during the right-hand movement is near the contralateral PMc; however, FC3 and FC5 during the left-hand movement are near the ipsilateral PMc, which was not assumed in the previous studies.

Although it is difficult to explain this observation based on current physiological knowledge completely, especially in terms of the activation of the ipsilateral side, there are several possibilities. There may be cases in which brain activity with different timing is observed near the left PMc for the right- and left-hand motor execution tasks even though the location is the same, or where different waveforms are observed, but these have not been identified in this study. Nevertheless, this study showed that it is possible to improve the BCI accuracy without relying on the somatotopic localization near the M1 region.

In general terms, the channels involved in classifying resting, left-handed, and right-handed motor executions in our BCI reflect activity near the PMc and M1 regions. The activity of these regions was reported to be activated during exercise from the studies of ERD (Pfurtscheller and Lopes da Silva, 1999), transcranial magnetic stimulation (TMS) (Aono et al., 2013), sensory evoked potentials (SEPs) (Starr and Cohen, 1985; Jiang et al., 1990; Seki et al., 2003; Seki and Fetz, 2012), and fMRI (Kasess et al., 2008; Ritter et al., 2009). In particular, BCI rehabilitation was reported to increase the effective connectivity from the affected SM1 to PMc and SMA around FC5, FC3, and FC1 with motor function recovery. These are the important parameters as an induced target of BCI rehabilitation, which is one of the BCIs for the training purpose (Biasiucci et al., 2018).

Considering studies on repetitive transcranial magnetic stimulation (rTMS) (Chen et al., 2003; Murase et al., 2005), SEPs (Starr and Cohen, 1985; Jiang et al., 1990; Seki et al., 2003; Seki and Fetz, 2012), fMRI (Kasess et al., 2008; Ritter et al., 2009), etc., it is desirable to improve the accuracy of such BCI by designing candidate generalized BCI features validated with cross-subjects for SM1 and other motor-related areas such as the SMA and PMc. However, there are no known successful cases of such an approach, owing to the feature design difficulty. This study adopted the different approach of data-driven design of a highly accurate BCI by end-to-end training of raw EEG data and discriminative labels, without assuming specific features and visualization of the model. The BCI model was tuned to reflect the influence of brain activity near the PMc.

### Features of Grad-CAM as a BCI Model Visualization Method for Residual-EEGNet

The BCI model has been visualized in a manner such that the CSP weight vector is displayed on a topography map (Jia et al., 2022a). The weight of CSP is calculated by maximizing the variance ratio between classes for each EEG channel, and the weight vector itself is visualized. The fundamental difference as a visualization method is that CAM and Grad-CAM can calculate and visualize feature maps for each input dataset. Because it is possible to visualize a collection of cases with only correct answers, omitting incorrect answers from the model, we consider that the findings obtained from the visualization results are highly reliable, which is the procedure followed in this study.

Note that the visualization of the CSP weight vectors includes both sides of the misclassified and correctly classified cases. Considering that it is also important to know how well our proposed model, which is the subject of the visualization, classifies the correct answers, i.e., how accurate it is, we compared it with models proposed in the previous studies (Schirrmeister et al., 2017; Lawhern et al., 2018) and also conducted an ablation study. The results showed that the model is more accurate than the compared three other models from the previous studies, thus ensuring a high degree of confidence in the visualization results. The Deep-ConvNet and Shallow-ConvNet models of previous studies were found to be as accurate or more accurate than the Filter Bank Common Spatial Pattern (FBCSP) (Schirrmeister et al., 2017). However, because CSP is often used in a preprocessing role, and its accuracy differs depending on the machine learning-based classification method of the main block, it is difficult to make comparisons in all cases.

We also consider that the reliability of the visualization results will vary depending on the method used to visualize the cross-validated models in this study. We distinguish between within-subject and cross-subject in cross-validation. Because within-subject validation is trained for each subject (Schirrmeister et al., 2017; Lawhern et al., 2018), we consider that the reliability of the model is not guaranteed for unknown subjects. This is because even if the model is trained to prevent overfitting in within-subject validation, the prevention of overfitting in cross-subjects is not guaranteed. Therefore, we consider that the latter type of model visualization, which guarantees the reliability of the model for unknown subjects, is more appropriate for finding neuroscientific knowledge for the target group of subjects. In this study, we adopted the latter method and confirmed that the reliability of the model for unknown subjects, i.e., the accuracy in the test data, is also higher than the accuracy of the conventional model.

In addition, the following is a supplemental explanation of the technical details. The model used for visualization is also often combined with a frequency filter in CSP to visualize the weight vector for a specific frequency, as shown by Jiao et al. (2020). That visualization method often captures phenomena such as ERD localized in SM1 (Zhang et al., 2021). However, in this study, we did not limit ourselves to frequency domain analysis assuming stationarity of the EEG, but used a model in which the features of the raw waveform were learned end-to-end to deal with the reliability issue in the methods. The visualization results showed that ipsilateral activity and frontal area (SMA or/and PMc), rather than phenomena such as ERD localized in SM1, were helpful for classification with high accuracy.

The advantage of Grad-CAM over CAM is that it can visualize the weights of the layers that contribute to the output of the predictive labels for each input dataset by calculating the variation in the output of the predictive labels of the final layer in response to the variation in the feature map, which is the feature gradient, and multiplying it by the feature map instead of the weight vector of the GAP in CAM. This allows visualization of CNN models not limited to GAP. In addition, we recognize that Grad-CAM can theoretically be applicable and used for CSP as long as the feature gradients can be calculated. Furthermore, models combining CSP and CNN have been reported (Yang et al., 2015). Therefore, we consider CSP as a candidate for a method to be combined as a preprocessing method rather than a model to be compared.

### Technical Limitations and Contributions to Future BCI Studies

In the recent years, it is being anticipated that BCI will be used in various fields, particularly in medicine. However, there is a need to improve its accuracy, the sophistication of the decoding targets, and the effectiveness of its use. The immediate goal of this study was to improve generalization performance without calibration. As this study is still a rudimentary attempt, the decoding targets were resting state and motor execution of left- and right-hand movements. It has not been validated on high-difficulty decoding tasks, which is a technical limitation at present.

In future work, we plan to further increase the number of layers in the model and try to improve the accuracy and difficulty of the decoding targets. Further technical improvements are also needed, such as increasing the size of the current dataset and data augmentation. If a motor execution task decoding model for healthy subjects, which is different from the patient model, can be constructed with sufficient generalizability through this study, it could result in an efficacy advantage over previous studies with respect to its application to rehabilitation for the purpose of restoring motor function in patients with stroke.

## Conclusion

This study confirmed the following:

1. The proposed BCI can be trained by end-to-end learning from raw EEG data.
2. A generalized model of motor classification of healthy people, who are the targets of BCI rehabilitation patients, was developed as a generalized BCI model for training.
3. The insertion of residual networks and the multilayer networks improved the accuracy of our BCI.
4. The proposed BCI contributed to the non-primary and primary motor-related areas in the Grad-CAM analysis.

In the future, an increase in the training data is expected to scale up the BCI model.

## Data Availability Statement

Publicly available datasets were analyzed in this study. This data can be found here: PhysioNet, https://physionet.org/, doi: 10.13026/C28G6P.

## Ethics Statement

Ethical review and approval was not required for the study on human participants in accordance with the local legislation and institutional requirements. Written informed consent for participation was not required for this study in accordance with the national legislation and the institutional requirements.

## Author Contributions

YF: conceptualization, methodology, software, formal analysis, investigation, data curation, writing—original draft, writing, reviewing, and editing, visualization, project administration, and funding acquisition. JU: methodology, validation, writing, reviewing, and editing, and supervision. Both authors contributed to the article and approved the submitted version.

## Funding

This study was partly supported by a Keio Leading-edge Laboratory of Science and Technology (KLL) Ph.D. Program Research Grant from Keio University.

## Conflict of Interest

YF is employed by the Information Services International-Dentsu (ISID) Ltd. JU is a founder and the Representative Director of the University Startup Company, Connect Inc. involved in the research, development, and sales of rehabilitation devices including brain-computer interfaces. He receives a salary from Connect Inc., and holds shares in Connect Inc. This company does not have any relationships with the device or setup used in the current study.

## Publisher's Note

All claims expressed in this article are solely those of the authors and do not necessarily represent those of their affiliated organizations, or those of the publisher, the editors and the reviewers. Any product that may be evaluated in this article, or claim that may be made by its manufacturer, is not guaranteed or endorsed by the publisher.
